# Surgeon General

**Published:** 1862-05

**Authors:** 


SURGEON GENERAL.—William A. Hammond M. D., has been *selected* by the President as Surgeon-General of the U. S. Army under the recent reorganization of the Medical Department. The profession will hear of the confirmation of this appointment with the most sincere gratification. No man could be selected, who so happily combines in his professional relations the confidence and esteem of both the Medical Staff of the army, and the profession of the country, as Dr. Hammond.

